# Targeting mTOR Signaling by Dietary Polyphenols in Obesity Prevention

**DOI:** 10.3390/nu14235171

**Published:** 2022-12-05

**Authors:** Yunyun Cao, Shuai Han, Han Lu, Yi Luo, Tianyi Guo, Qi Wu, Feijun Luo

**Affiliations:** 1 Hunan Provincial Key Laboratory of Grain-Oil Deep Process and Quality Control, Hunan Provincial Key Laboratory of Forestry Edible Resources Safety and Processing, Hunan Provincial Key Laboratory of Processed Food for Special Medical Purpose, College of Food Science and Engineering, Central South University of Forestry and Technology, Changsha 410004, China; 2 Department of Clinic Medicine, Xiangya School of Medicine, Central South University, Changsha 410008, China

**Keywords:** dietary polyphenols, obesity, mTOR, signal pathway

## Abstract

Dietary polyphenols can be utilized to treat obesity and chronic disorders linked to it. Dietary polyphenols can inhibit pre-adipocyte proliferation, adipocyte differentiation, and triglyceride accumulation; meanwhile, polyphenols can also stimulate lipolysis and fatty acid β-oxidation, but the molecular mechanisms of anti-obesity are still unclear. The mechanistic target of rapamycin (mTOR) is a protein kinase that regulates cell growth, survival, metabolism, and immunity. mTOR signaling is also thought to play a key role in the development of metabolic diseases such as obesity. Recent studies showed that dietary polyphenols could target mTOR to reduce obesity. In this review, we systematically summarized the research progress of polyphenols in preventing obesity through the mTOR signaling pathway. Mechanistically, polyphenols can target multiple signaling pathways and gut microbiota to regulate the mTOR signaling pathway to exert anti-obesity effects. The main mechanisms include: modulating lipid metabolism, adipogenesis, inflammation, etc. Dietary polyphenols exerting an anti-obesity effect by targeting mTOR signaling will broaden our understanding of the anti-obesity mechanisms of polyphenols and provide valuable insights for researchers in this novel field.

## 1. Introduction

Obesity is a public health issue with a high mortality rate, and can contribute to cardiovascular disease, type 2 diabetes, and several malignancies [1]. Most obesity and overweight have environmental factors or genetic interactions causations. Environmental factors are mainly divided into sedentary jobs, technology advancements, and sufficient unhealthy foods [2]. These influencing factors or other factors can lead to an overabundance of body fat by an energy imbalance between consumed and burned calories. Currently, losing weight, medical treatment, and bariatric surgery are the main treatments for obese population. However, the major problem with severe obesity lies in the short-term side effects and complications of conventional treatment [3].

Many chronic disorders, including obesity, can be prevented and treated in part by dietary modification. Specific dietary treatments for obesity improve weight reduction and reduce weight regain [4]. Due to their potent anti-obesity effects and low toxicity, dietary polyphenols have attracted much attention in the prevention and treatment of obesity in recent years. Studies showed that dietary polyphenols can reduce the viability of adipocytes and proliferation of preadipocytes, suppress adipocyte differentiation and triglyceride accumulation, stimulate lipolysis and fatty acid β-oxidation, and reduce inflammation by regulating multiple signal pathways [5,6]. This suggests that modulating lipid-metabolism-related pathways can exert a lipid-lowering function.

The mechanistic target of rapamycin (mTOR), which can be activated by stimulants to preserve the integrity of cellular homeostasis, is the important molecule in the regulation of cell growth [7,8]. As a major growth controller, mTOR is activated whenever nutrient availability or environmental signals fluctuate to control the processes required for cell growth and proliferation [9]. mTOR controls cell growth by activating anabolic processes such as protein, lipid, and nucleotide synthesis, stimulating energy metabolisms such as glycolysis and glutaminolysis, and inhibiting catabolic processes [9,10,11]. The deregulation of anabolism and catabolism occurs in human disease, such as obesity, type 2 diabetes, and cancer. The over-activation of mTOR plays an important role in adipogenesis. A possible major target for the prevention and treatment of obesity is mTOR because of its central involvement in cell growth and proliferation. 

The function of dietary polyphenols and mTOR signaling in the prevention of obesity was systematically summarized in this paper. In order to prevent obesity, the molecular mechanisms by which dietary polyphenols target the mTOR signaling pathway were examined. The application prospects and difficulties in this area were also covered. This will give fresh suggestions for using dietary polyphenols to prevent obesity in its early stages.

## 2. Dietary Polyphenols’ Impact on Obesity

Polyphenols are a prominent class of complex and widespread plant metabolites in the human diet [12]. The human intake of plant-derived foods contains thousands of structurally different polyphenols, including nuts, cocoa, dark berries (especially grapes and cherries), tea beverages (approximately 89 mg and 102 mg of polyphenols per 100 g of green and black tea, respectively), grains, and red wine [13,14]. In the same volume (125 mL), a glass of red wine contains approximately 5 mg polyphenols, which is more than the dosage in a cup of coffee [15]. The main types of polyphenols include flavonoids, phenolic acids, stilbenes, and lignans [16]. Polyphenols in the diet have been demonstrated to benefit a number of pathological diseases [17]. This is due to the fact that different polyphenols have different sugar units and acylated sugars at various positions in their backbone. Polyphenols have been produced as foods or medications with positive health effects due to their potent biological functions, lack of adverse effects, and wide availability. Numerous studies have shown that a high-polyphenol food intake is closely related to human obesity and weight control [14]. Table 1 [18,19,20,21,22,23,24,25,26,27,28,29,30,31,32,33,34,35,36,37,38,39,40,41,42,43,44,45,46,47,48,49] summarizes the representative anti-obesity polyphenols and their dietary sources.

The biological functions of flavonoids depend on their structural differences and glycosylation patterns. Catechins, anthocyanins, and quercetin are the main subtype flavonoids with anti-obesity effects. Approximately 63% of green tea catechins are (-)-epigallocatechin gallate (EGCG), making it the most prevalent catechin [50]. Studies have shown that EGCG can interfere with multiple signal transduction pathways to exert an anti-obesity effect [5]. In the high-fat diet-induced obesity model of mice, EGCG has positive effects on obesity-related markers and improves glucose homeostasis [31]. In 3T3-L1 adipocytes, EGCG inhibited preadipocyte differentiation by regulating the expression of key transcription factors in the early stages of differentiation, such as peroxisome proliferator activator receptor γ (PPARγ) and CCAAT/enhancer binding protein α (C/EBPα) [51]. By interfering with the cell cycle during the clonal development of 3T3-L1, EGCG also inhibited cell proliferation [52]. For obesity-induced chronic inflammation, EGCG can down-regulate inflammatory markers and reduce oxidative stress levels [53]. Resistin is an adipocyte-derived inflammatory adipokine. EGCG can inhibit the expression of the resistin gene, thereby reducing the risk of insulin resistance [54]. However, the molecular targets and signaling pathways of EGCG intervention are different in different experimental models, and not all studies have shown an obvious effect of EGCG on obesity [55].

Anthocyanins are mainly responsible for the color in fruits, vegetables, and grains [56]. For diet-induced obesity, anthocyanins can inhibit adipogenesis, lipid metabolism, and adipose inflammation [57]. One of the causes of the development of obesity and metabolism-related diseases may be changes in gut bacteria. In a high-fat diet (HFD)-induced obese model, body weight and steatosis scores were reduced after pomegranate peel anthocyanins treatment [33]. This effect lies in pomegranate peel anthocyanins reducing the ratio of Firmicutes or Bacteroidetes and increasing the abundance of *Akkermansia muciniphila*. In 3T3-L1 adipocytes, cyanidin-3-O-β-glucoside (C3G) inhibited adipocyte lipolysis [58]. Anthocyanins from fruit inhibited adipogenesis and adipocyte differentiation in cells [59]. The level of the obesity-associated inflammatory factor interleukin-6 (IL-6) was significantly reduced in participants who regularly consumed anthocyanins [60]. There is increasing evidence that anthocyanins have anti-obesity effects in different obesity models through multiple pathways. 

Quercetin is a flavonoid with several biological functions, some of which are found in leafy vegetables, fruits, and tea. In muscle-derived mesenchymal progenitors, quercetin inhibited adipogenesis and fibrosis to prevent the loss of muscle mass [61]. Key adipogenesis factors can be inhibited by quercetin to inhibit adipogenesis [62]. In HFD-induced mice, quercetin increased the glucose uptake in adipose tissue and inhibited adipose tissue macrophage infiltration [63]. The browning of white adipose tissue may contribute to quercetin’s plasma triglyceride-lowering action [64]. For overweight and obese women, 12 weeks of supplementation with quercetin-rich onion skin extract significantly reduced body fat percentage and plasma adiponectin levels [65]. Quercetin-rich extracts showed anti-obesity effects in preadipocytes of obese rats by inhibiting preadipocyte differentiation and preventing adipogenesis [66]. Additionally, quercetin significantly reduced the inflammatory state of visceral adipose tissue in genetically obese rats [67]. So far, a growing body of literature suggests that quercetin regulates lipid metabolism through different mechanisms.

Gallic acid and chlorogenic acid are benzoic acid derivatives and cinnamic acid derivatives that are more studied. They are widely found in daily diet, including berries, tea drinks, and some vegetables. Studies had shown that oral gallic acid can improve liver steatosis and reduce the body weight and plasma insulin level in mice fed with HFD. It was found that oral gallic acid significantly inhibited ACC and FASN mRNA levels in obese mice by detecting genes related to steatosis in mouse liver [68]. In addition to improving obesity, gallic acid has also been found to improve insulin signaling and reduce inflammation and oxidative stress [69]. The supplementation of chlorogenic acid can also improve obesity. Chlorogenic acid played an anti-obesity role mainly by reducing food intake and increasing energy consumption in mice [70]. In HepG2 cells, chlorogenic acid inhibited the mRNA and protein levels of HMGCR to regulate cholesterol metabolism [71]. Phenolic acids have attracted great attention due to their various biological tasks.

Resveratrol is a naturally occurring polyphenolic substance that is mostly present in grapes, red wine, and some berries. A recent study showed that resveratrol supplementation significantly affects lipid regulation in obese patients [47]. In diet-induced obese rats, resveratrol also affected blood lipids and inflammatory responses to reduce the rat body weight [72]. After receiving resveratrol treatment, the obese mice’s gut microbiota composition was drastically changed [73]. Brown adipose tissue (BAT) has many beneficial functions in obesity. Resveratrol may promote the release of myokines and adipokines and function as an activator of fat browning [74]. Surprisingly, resveratrol could affect the metabolism of the offspring of obese rats [75]. In conclusion, resveratrol can prevent or treat obesity for human benefits.

In animal or cell models, these dietary polyphenols have been demonstrated to reduce obesity when taken as supplements (pure compounds). In fact, the metabolism, transformation, and physiological concentration of polyphenols are rarely considered by researchers [76]. The intake of dietary polyphenols in the human diet is usually low. Despite significant limitations, some epidemiological and clinical studies have evaluated the dietary polyphenol intake. The average person (including children, adults, and seniors) consumes roughly 900 mg of total polyphenols per day [77]. Another problem with dietary polyphenols is the intake of compounds by the test organisms. It is well known that polyphenols have a low bioavailability, especially the absorption of polyphenols by humans, which is often poor. The interaction with other nutrients, liver metabolism, and intestinal microbiota are the main influencing factors [78]. Using proteins or liposomes to encapsulate or form polymers with other compounds is a common method of improving the stability and absorption of polyphenols [79].

Taken together, dietary polyphenols have a tremendous deal of promise in reducing obesity. In obesity, dietary polyphenols can promote lipid metabolism, induce white adipose tissue browning, and inhibit preadipocyte differentiation and adipogenesis. In addition, dietary polyphenols may alleviate obesity complications such as cardiovascular disease and cancer. Dietary polyphenols can also modulate gut microbiota to mitigate the development of obesity. However, growing evidence indicates that the anti-obesity effects of dietary polyphenols are involved in the mTOR signaling pathway. Targeting mTOR signaling by dietary polyphenols is a novel mechanism in obesity prevention.

## 3. mTOR Functions

mTOR is a conserved serine–threonine protein kinase that senses and integrates various extracellular and intracellular signals, such as cell growth factors and different nutrients, to cellular and organismal responses [80,81]. It is essential for several biological activities, including cell proliferation, growth, and autophagy [80]. The dysregulation of mTOR occurs in many pathological conditions, including type 2 diabetes, aging, cancer, and obesity.

mTOR belongs to the phosphoinositide 3-kinase (PI3K)-related kinase family because they share similar catalytic domains [80]. mTOR forms two distinct complexes called mTOR complex 1 (mTORC1) and 2 (mTORC2) by binding to several proteins. Sec-13, DEPTOR, and the Tti1/Tel2 complex are present in mTORC1 and mTORC2 [82,83]. By comparison, mTORC1 contains specific regulators RAPTOR and PRAS40 [84,85,86], whereas mTORC2 contains RICTOR, mSin1, and PROCTOR1/2 [87,88,89].

Since the substrate preferences are different for the two kinase complexes, the cellular functions modulated by them are also different. Studies show that mTORC1 is a dimer structure with multiple domains. mTOR and raptor regulate mTORC1 activity by phosphorylating some residues. mTORC1 is activated as a master regulator by environmental signals, including nutrient and growth factor signals, to coordinate substance synthesis, inhibit autophagy, and stimulate cell growth. Growth hormones and certain nutrients, such as amino acids, can activate mTORC1 in lysosomes [90]. Growth factors activate the PI3K-PDK1-AKT signaling pathway and inhibit the TSC complex [91,92]. The complex can make Ras homologue enriched in brain (RHEB), thereby inhibiting mTOR [93]. The RHEB activation of mTORC1 is affected by its ubiquitination [94]. Mitogen-activated protein kinase (MAPK) promotes raptor phosphorylation to activate mTORC1. Cellular energy has an impact on mTORC1 in addition to growth factors. AMP-dependent kinase (AMPK) acts as an energy sensor, activated by low cellular energy, and downregulates mTORC1 by TSC [95]. Amino acids can regulate mTORC1 through multiple amino acid sensors and protein mechanisms [96]. Lipid, cholesterol, and purine nucleotide levels [97] can also modulate mTORC1 activity. mTORC1 mediates different downstream target proteins to control catabolism and synthesis metabolism, such as the inhibitory eIF4E-binding protein (4EBP1), ribosomal protein S6 kinase 1 (S6K1), and sterol regulatory element binding proteins (SREBPs) [98,99]. Compared with mTORC1, the upstream regulatory factors of mTORC2 are largely uncertain, except for the growth factor/PI3K signal axis. The PI3K signal activates mTORC2 by encouraging the binding of the kinase complex to the ribosome, although its mechanism is unclear [100]. Although mSin1 binds directly to rictor, it may not reflect the function of mTORC2 alone [101]. Phosphatidic acid (PA) is also associated with mTORC2 activation. mTORC2 phosphorylates the kinase complex, thereby regulating cell survival, metabolism, apoptosis, growth, and proliferation [102]. The downstream complex contains PKA, PKG, and PKC, which are a part of the AGC-kinase family [103,104]. In addition to controlling organismal growth and homeostasis, the mTOR signaling pathway has been linked to an increasing variety of clinical diseases, including obesity.

## 4. mTOR and Obesity

**Figure 1:**
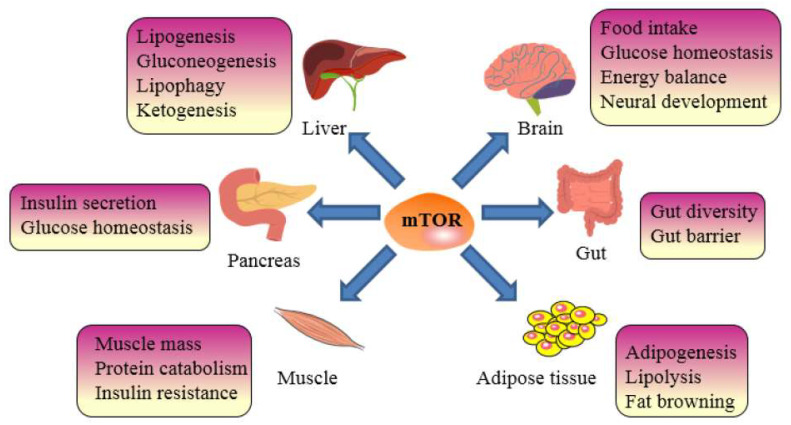
The mechanisms of mTOR signal pathway participating anti-obesity effect.

Previous research indicates that obesity can trigger a chronic excessive activation of mTOR activity in multiple tissues [105]. We focused on the role of the mTOR complex in energy homeostasis and metabolism in vital metabolic tissues such as the brain, gut, adipose tissue, liver, pancreas, and skeletal muscle in obese patients (see Figure 1).

The brain’s hypothalamus, a crucial region, combines messages to regulate energy balance. mTORC1 might promote orexin Y expression in the hypothalamus and reduce food intake via S6K1 [106]. The pathway of mTORC1 activation by amino acids and neurotrophic growth factors is mainly concentrated in the TSC1-TSC2 complex. The over-activation of mTORC1 may inhibit mTORC2 [107]. In the hypothalamus of HFD rats, the methylation of Tsc1-mTOR signaling may alleviate obesity by upregulating the expression of lipid-metabolism-related genes [108]. In the hypothalamus of PGC-1β-ablated mice, PGC-1β coordinates the mitochondrial biogenesis and function with the constitutive activation of the mTORC1 pathway [109]. The gut microbiota and its metabolites play important roles in host metabolism and immunity [110]. Diseases such as obesity, diabetes, and cancer may occur as a result of dynamic changes in the gut microbiota. The proportion of Bacteroidetes and Firmicutes is related to the lymphatic structure, immune system, food intake, and metabolism in obese [111]. An excessive energy intake may lead to metabolic disturbances and the over-activation of mTORC1, which is often accompanied by changes in the gut microbiome [110,112]. Antidiabetic effects are associated with alterations in the gut microbial composition (by an increase in the *Akkermansia* spp. population) in metformin (mTORC1 inhibitor)-treated mice on a high-fat diet [113]. Additionally, dyslipidemia in obese animals is associated with gut microbial metabolites (short-chain fatty acids, bile acids) and mTORC1 expression [114,115]. In another study, the inhibition of mTORC2 signaling prompted changes in the composition of the gut microbiota in HFD mice [116]. Among them, Lactococcus, Clostridium XI, Oscillibacter, and Hydrogenoanaerobacterium are associated with the obesity phenotype [116]. mTOR signaling plays a key role in the adipogenesis and maintenance of fat tissues [117,118]. mTORC1 is involved in the growth of normal adipose tissue and the transformation of two types of adipose tissues (white adipose tissue and brown fat) in vivo and in vitro [119]. Studies have also shown that mTORC1 is associated with fat browning [120]. Adipogenesis is prevented by mTORC1 inhibition, and adipocyte maintenance is compromised [121], whereas mTORC1 over-activation promotes adipogenesis [122]. Raptor knockout mice have a reduced adipose tissue and improved insulin sensitivity attributable to an enhanced energy expenditure due to mitochondrial uncoupling [123]. Rictor knockout studies carried out in animals indicate that mTORC2 controls whole-body growth and regulates the size of fat cells and organs [104]. In addition, mTORC2 in adipose tissue is correlated to adipocyte differentiation and white adipose tissue browning [124,125]. In brown adipocytes, a reduced mTOR activity stimulates mitophagy, which is essential for thermogenesis [126]. In 3T3-L1 adipocytes, mitochondrial dysfunction may attenuate insulin signaling through the oxidation of Rictor in the mammalian target of mTORC2 [127]. As an important lipid metabolic organ, the liver is the main place for adipogenesis and lipid oxidation, and impaired lipid metabolism in the liver is closely related to obesity [128,129]. mTORC1 enhances adipogenesis through the positive regulation of SREBPs, which belongs to transcription factor and controls lipid synthesis [99,130]. The phosphatidic acid phosphatase Lipin-1 is negatively regulated by mTORC1 to regulate SREBPs [131]. In addition, the balance between free and bound mTORC1 and Raptor is also an important regulatory mechanism for liver lipid accumulation [65]. In the liver, mTORC2 regulates the activity of glucokinase and SREBP1c via AKT phosphorylation, thereby regulating glucose and lipid metabolism [132,133]. Additionally, it is widely known that mTORC2 controls gluconeogenesis and adipogenesis by way of a number of transcription factors. Under nutrient overload conditions, an increased phosphorylation of mTOR may be associated with mitochondrial dysfunction [134]. Dysregulated AKT-mTOR signaling reduces mitochondrial function, leading to liver injury in obese mice [135]. mTOR activity has significant effects on the host by affecting the growth, proliferation, cell mass, and glucose homeostasis of pancreatic β or α cells. The activation of mTORC1 results in β-cell hypertrophy, and, conversely, mice lacking mTOR or Raptor have a reduced β-cell mass [136,137]. The role of mTOR in glucose homeostasis is uncertain. The transient activation of mTORC1 improves glucose metabolism, whereas a sustained activation impairs the pancreatic β-cell quality and function [138]. Skeletal muscle is an important organ for systemic metabolism. Unlike other organs, alterations in mTORC1 signaling in skeletal muscle have differential effects on systemic metabolism [139]. The continued activation of mTORC1 activates autophagy, which reduces muscle mass in mice [140]. In the muscle of mice specifically deficient in TSC1, mTORC1 is activated to regulate glucose uptake [139]. mTOR activation increases the muscle insulin sensitivity and insulin signaling in obese women [141]. In obese mice, an increased phosphorylation of mTOR signaling alleviates skeletal muscle atrophy. In conclusion, mTOR signaling plays an important role in major metabolic organs (hypothalamus, gut, adipose tissue, liver, pancreas, and skeletal muscle) in obese hosts.

## 5. mTOR Targeting by Dietary Polyphenols in Obesity

**Figure 2:**
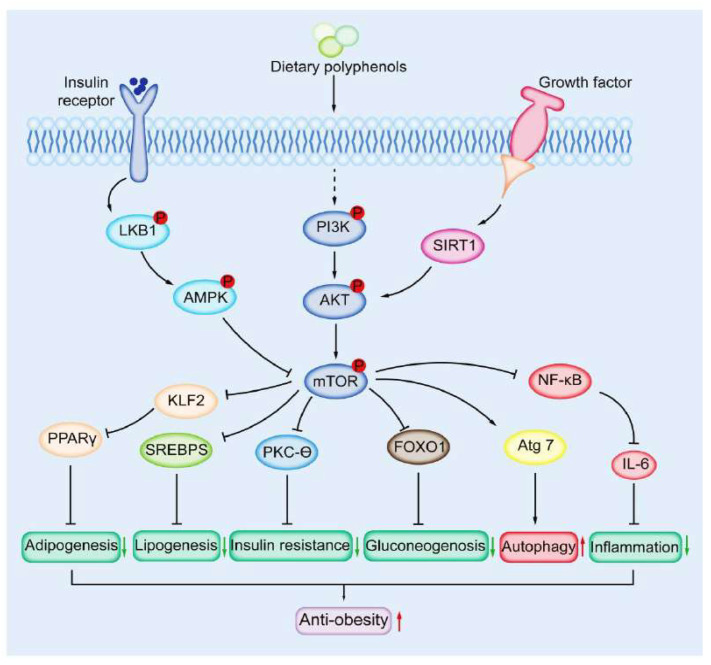
Targeting mTOR signaling by dietary polyphenols in obesity prevention. Note: “↑” indicates increase and “↓”indicates decrease.

mTOR, as a key target to control energy metabolism, is an important protein for preventing and improving obesity. Publications indicate that dietary polyphenols exert anti-obesity effects via targeting mTOR. Here, the progress and molecular mechanisms are systematically reported in Table 2 [142,143,144,145,146,147,148,149,150,151,152,153,154,155,156,157,158,159,160,161,162,163,164] and Figure 2.

AMPK: AMP-dependent kinase; mTOR: mechanistic target of rapamycin; PI3K: phosphoinositide 3-kinase; PPARγ: peroxisome proliferator activator receptor gamma; SREBPs: sterol regulatory element binding proteins.

### 5.1. Lipid Metabolism

The regulation of lipid metabolism is a common mechanism for the treatment of obesity. Lipid metabolism mainly includes the biosynthesis and degradation of lipids. Metabolic dysregulation associated with obesity may lead to dyslipidemia and lipid deposition [165].

A large body of evidence suggests that dietary polyphenols can regulate intracellular lipid metabolism by targeting the mTOR signaling pathway. For example, fisetin is a flavonoid found in fruits and vegetables. The results show that the addition of fisetin (10 μM) reduced intracellular lipid accumulation during 3T3-L1 adipocyte differentiation. Western blot analysis showed that the phosphorylation levels of mTOR and its downstream molecule S6K were inhibited by fisetin. Consistent with the quantitative PCR analysis, C/EBP α and GLUT4 protein levels were decreased after fisetin treatment. The addition of rapamycin (mTOR inhibitor) proved that mTOR signaling was involved in regulating GLUT4 gene expression in adipocytes. This means that fisetin inhibited GLUT4-mediated glucose uptake by inhibiting mTOR signaling, thereby inhibiting the accumulation of intracellular lipids [149]. Moreover, genistein is a widely distributed dietary phytoestrogen. Genistein (25 μM) can improve fatty acid β-oxidation in HepG2 cells by enhancing peroxisome proliferator-activated receptor α (PPARα) and carnitine palmitoyl transferase-I (CPT-1) expression levels [151]. This is related to the inhibition of protein phosphorylation levels of AKT and mTOR by GEN. Sulforaphane has positive effects on lipolysis in adipocytes (10 μM) and autophagy in the epididymis of mice (30 mg/kg body weight) [164]. Sulforaphane was shown to lower the autophagy of adipocytes using autophagic flux measurement and Western blotting measurement by increasing the protein expression of lipidated LC3 (LC3-II). The AMPK-mTOR-ULK1 signaling pathway is considered to be the key mechanism for sulforaphane to exert lipophagocytic activity. Anhydroicaritin is a prenylated flavonoid naturally present in the Chinese herbal medicine Epimedium. The mechanism of anhydroicaritin improving diet-induced obesity and hyperlipidemia in C57BJ/6L mice was studied and verified in vitro. Anhydroicaritin alleviated the weight gain of mice induced by a western diet in a dose-dependent manner. Anhydroicaritin could be used as a specific molecule to target and inhibit the activation of SREBPs through the LKB1/AMPK/mTOR pathway, thereby improving diet-induced obesity [142]. Additionally, proanthocyanidins are the most abundant polyphenols in dietary sources. In the high-fat-diet-induced obesity model, mice received an intragastric administration of grape seed proanthocyanidin extract (200 mg/kg/d) for six weeks, and their weight gain and lipid metabolism disorder were improved without adverse reactions [150]. The possible mechanism of extract to improve dyslipidemia was to reduce the mRNA level and protein expression of m-TOR and FOXO1 and promote autophagy flux. Lipophagy is a form of selective autophagy that degrades lipid droplets in adipose tissue and liver. In palmitate-induced pancreatic β cells, kaempferol treatment activated lipophagy via the AMPK/mTOR signaling pathway and reduced intracellular lipid deposition [153]. Compared with kaempferol-treated cells in the presence of palmitate, the cells co-treated with kaempferol and compound C (AMPK inhibitor) showed an approximately 2- and 3.5-fold increase in lipid and triglyceride content, respectively (*p* < 0.05). Taken together, dietary polyphenols target mTOR to exert protective effects, deceasing lipid accumulation and promoting fatty acid β-oxidation and lipolysis.

### 5.2. Adipogenesis and Lipogenesis

The process of the proliferation and differentiation of adipocyte precursor cells into mature adipocytes is defined as adipogenesis. Controlling adipogenesis and lipogenesis may be the key to obesity treatment.

In 3T3-L1 preadipocytes, fisetin inhibited AKT-mTORC1 signaling and adipogenesis-related genes in a dose-dependent manner, thereby inhibiting adipocyte differentiation [148]. Likewise, Kaempferol (7.5–30 μM) could block the phosphorylation of AKT and mTOR, thus inhibiting the accumulation of lipids during preadipocyte differentiation [152]. Quercetin treatment could activate m-TOR and p70S6K, which were associated with inhibiting adipogenesis, thus reducing lipid accumulation in the cells [159]. Pentamethylquercetin is a methylated quercetin derivative with a higher bioavailability. After 63 days of pentamethyl quercetin (20 mg/kg) in obese mice, the body weight and adipose tissue weight of the mice were significantly reduced. RT-PCR analysis revealed that the expressions of PPARγ, SREBP1, FAS, and other adipogenic genes in epididymal adipose tissue were changed significantly. Using an SIRT1 inhibitor to block SIRT1 activation, it was found that mTOR mRNA expression was increased. These results suggest that pentamethylquercetin inhibits visceral adipogenicity by inhibiting Sirt1-mediated mTOR and adipogenesis signaling pathways [157]. Penta-O-galloyl-α-D-glucose (α-PGG) is a hydrolyzable tannin compound. α-PGG can induce preadipocyte cycle arrest through mTOR/p21 to prevent adipogenesis in vitro [158]. In the 3T3-L1 preadipocyte differentiation model, oligonol inhibited the AKT/mTOR signaling pathway and down-regulated the expressions of lipid biosynthesis-related genes such as PPARγ, CEBPα, and CEBPδ, which prevented 3T3-L1 preadipocyte differentiation and an adipogenic effect [155]. In a high-fat-diet-induced obesity mouse model, 20 or 200 mg/kg bw of lychee fruit extract (oligonol) was administered by gavage for 6 weeks, and the results showed that oligonol prevented weight gain in mice and decreased mTOR activity. Oligonol could reduce the lipid content in liver cells, thereby inhibiting de novo lipogenesis [154]. The inhibition of the AKT/mTOR pathway by capsaicin (200 μM) indirectly inhibited SREBP-1c, thereby inhibiting de novo lipogenesis [145]. In HepG2 cells, cells treated with betulinic acid (1, 2, 3, and 4 μg/mL) for 48 h could reduce intracellular lipid accumulation. After IR or IGF1 stimulation, the mTOR signaling pathway was inhibited. Betulinic acid inhibited de novo lipogenesis by inhibiting IR or IGF1 signaling and the downstream mTOR pathway [144]. All of the evidence suggests that many dietary polyphenols can modulate adipogenesis and lipogenesis via targeting the mTOR pathway.

### 5.3. Insulin Dysregulation

Insulin dysregulation mainly includes hyperinsulinemia and insulin resistance. Obesity often causes insulin resistance, leading to hyperglycemia, which, in turn, affects the secretion of insulin by the pancreas to maintain glucose homeostasis. This can lead to hyperinsulinemia and an absolute increase in circulating insulin. Obesity alters systemic metabolism and increases the risk of insulin resistance in metabolic tissues. Rapamycin or resveratrol prevented weight gain in mice on a high-fat diet, but this could have long-term side effects at high doses [160]. Interestingly, a low-dose combination of rapamycin and resveratrol (<10 μM) prevented hyperinsulinemia and obesity in HFD mice without inhibiting the mTOR pathway [160]. In palmitate-induced hepatocytes, oligonol (a polyphenolic polymer) showed a positive effect on insulin resistance [156]. Western blot analysis showed that oligonol significantly inhibited the phosphorylation of mTOR and S6K. Oral resveratrol prevented diet-induced glucose intolerance in obese mice, which was associated with changes in mTOR complex 2 activity [116]. Anthocyanins from purple corn husk improved tumor necrosis factor-α-induced insulin resistance in 3T3-L1 adipocytes, possibly associated with a reduced hyperglycemia in the metabolic syndrome [143]. The results of the insulin receptor fluorescence array show that anthocyanins reduced the phosphorylation level of mTOR and p70S6K [143]. Insulin resistance is associated with obesity and high plasma free fatty acid levels. In palmitate-treated L6 skeletal muscle cells, mTOR phosphorylation levels were increased and insulin-stimulated GLUT4 glucose transporter membrane levels and the glucose uptake were decreased [163]. Resveratrol treatment relieved this insulin resistance [163]. These results suggest that dietary polyphenols can regulate insulin dysregulation through the mTOR pathway.

### 5.4. Gut Microbiota and Inflammation

Intestinal microbiota play an important role in the development of metabolic diseases, while supplementing dietary polyphenols can help to restore the imbalance of intestinal microbiota [166]. The combination of resveratrol and quercetin restored the gut microbiota dysbiosis of obese rats caused by a high-fat diet [167]. Tea polyphenols and their derivatives could effectively inhibit obesity and related metabolic disorders by regulating intestinal micro-ecology [168]. Oral resveratrol (a specific inhibitor of the mTOR complex 1 prevented glucose intolerance and fat accumulation in HFD-fed mice. The species abundance of *Lactococcus*, *Clostridium XI*, and *Oscillibacter* decreased after resveratrol treatment [116]. In the state of obesity and its related diseases, a persistent, low-grade inflammatory reaction can usually be detected. In differentiated preadipocytes, resveratrol promoted Sirt1 and AKT2 interaction and reduced the level of mTOR complex 1, thereby inhibiting adipose inflammation [161]. These data indicate that the diet can modulate gut microbiota and inhibit inflammation, which is involved in the activation of mTOR.

### 5.5. Other Biological Functions

Obesity, an extremely complex disease, has a series of continuous complications. In many neurodegenerative diseases, such as Alzheimer’s disease, defects in autophagy activation are prevalent, while mTORC1 signaling is considered to be the most important regulator of autophagy. Obesity is considered as one of the risk factors for the development of Alzheimer’s disease. Resveratrol activates AMPK in the prefrontal cortex and hippocampus of obese mice, inhibits mTORC1, and activates autophagy, thereby clearing away amyloid protein-β peptide [162]. The incidence of colorectal cancer is associated with the incidence of obesity. In an azoxymethane-treated mouse model, the combination of curcumin and salicylic acid could significantly inhibit the activation of PI3K/AKT/mTOR signaling and prevented colon tumor development in high-fat-diet (HFD) mice [146]. In human experiments, obese women took green tea extract supplements for 8 weeks without significant changes in body weight and fat mass. However, EGCG upregulated the expression of Rictor (an essential subunit of the mTORC2 complex) [147]. All data suggest that mTOR is a vital target of polyphenols and plays a very important role in obesity prevention.

## 6. Conclusions

Obesity and its complications contribute to many diseases and seriously damage people’s health. Investigations indicate that dietary polyphenols have a positive role in preventing obesity and obesity-related chronic diseases. Polyphenols can modulate multiple signal pathways to inhibit obesity; meanwhile, polyphenols can also regulate gut microbiota and produce metabolic productions to prevent obesity. mTOR plays a key role in glucose metabolism and lipid metabolism. Regardless of whether polyphenols modulate signal pathways or gut microbiota, mTOR seems to be the important target of the anti-obesity effect. Most studies focus on the positive effects of dietary polyphenol supplements on obesity in animal or cell models. Since these dietary polyphenols are generally regarded as safe, it may be necessary in the future to conduct more human clinical trials to test the hypothesis that the phytochemicals might be useful in controlling obesity. So far, it is still unclear how polyphenols affect signaling pathways and key target genes by targeting mTOR. Why do some polyphenols affect mTOR and others not affect the pathway? Can different structural polyphenols affect mTOR activation? Or can all polyphenols modulate mTOR activation? If different polyphenols can regulate mTOR, why are the downstream targets different? All issues need further investigation in the future. The gut–brain axis (GBA) plays a very important role in the pathophysiology of obesity, which is mediated by metabolic, endocrine, neural, and immune system mechanisms. Polyphenols targeting mTOR modulate inflammation, insulin resistance, and so on, which means that polyphenols affect obesity through the gut–liver–brain axis by targeting mTOR. Unfortunately, no publication has investigated polyphenols targeting mTOR participating in the gut–liver–brain axis; for example, can blocking the mTOR pathway affect appetite or inflammation of the brain in the polyphenols-induced anti-obesity model? Overall, dietary polyphenols targeting mTOR signaling in the prevention of obesity is beneficial for humans; however, the specific mechanism remains to be further explored.

## Figures and Tables

**Table 1:** Types and food sources of dietary polyphenols with anti-obesity effects.

Polyphenols	Subtype	Major Food Sources	References
Quercetin	Flavonols	apple, berries, grape, red onions, broccoli, black tea, green tea, pepper, red wine, and tomato	[18]
Kaempferol	Flavonols	spinach, kale, dill, chives, and green leafy vegetables	[19]
Myricetin	Flavonols	apple, peach, orange, pineapple, and sweet potato	[20]
Isorhamnetin	Flavonols	dill weed, sea buckthorn berries, and kale onions	[21]
Fisetin	Flavonols	strawberries, apple, persimmons, grape, onions, and cucumbers	[22]
Luteolin	Flavones	parsley, shiso, celery, pepper, broccoli, and thyme	[23]
Apigenin	Flavones	thyme, cherries, tea, olives, broccoli, legumes, the leafy herb parsley, and dried flowers of chamomile	[24]
Acacetin	Flavones	propolis, chrysanthemum, and galangal	[25]
Naringenin	Flavanones	tomatoes, cocoa, cherries, citrus paradise, citrus sinensis, bergamot, and citrus fruit	[26]
Hesperetin	Flavanones	tangerines, oranges, lemons, and citrus fruit	[27]
Eriodictyol	Flavanones	lemons, peanut, vegetables, and fruits	[28]
Catechin	Flavanols	tea, broad beans, red wine, grape, strawberries, and apricots	[29]
Epicatechin	Flavanols	tea, rosa roxburghii tratt, cocoa, dark chocolate, berries, and apple	[30]
Epigallocatechin-3 -gallate	Flavanols	tea leaves, cocoa products, pome fruits, prune juice, and broad bean pod	[31]
Proanthocyanidins	Flavanols	barley, hops, tea, maize, apple, grape, strawberries, cocoa, almonds, cinnamon, peanuts, and vegetables	[32]
Cyanidins	Anthocyanins	beans, fruits, vegetables, and red wines	[33]
Delphinidins	Anthocyanins	pigmented fruits and vegetables	[34]
Malvidins	Anthocyanins	wine, grape, pomegranate, and pigmented fruits	[35]
Genistein	Isoflavones	soybean and leguminous plants	[36]
Daidzein	Isoflavones	soy and soy-derived products	[37]
Formononetin	Isoflavones	soybean, astragalus mongholicus, and licorice	[38]
Gallic acid	Derivatives of benzoic acid	chestnuts, tea, wine, grapes, berries, and other fruits	[39]
Vanillic acid	Derivatives of benzoic acid	angelica sinensis and green tea	[40]
Protocatechuic acid	Derivatives of benzoic acid	mushrooms, olives, apple, red wine, and grape	[41]
Ferulic acid	Derivatives of cinnamic acid	eggplants, tomatoes, spinach, beer, peanuts, and grains	[42]
p-Coumaric acid	Derivatives of cinnamic acid	apples, pears, strawberries, other berries, peanuts, rye bran, and red wine	[43]
Caffeic acid	Derivatives of cinnamic acid	apples, pears, berries, blueberry, plum, eggplant, carrot, and coffee	[44]
Chlorogenic acid	Derivatives of cinnamic acid	coffee beans, tea, peaches prunes, eggplants, and vegetables	[45]
Sinapic acid	Derivatives of cinnamic acid	vegetables and whole grains	[46]
Resveratrol	Stilbenes	grapes, berries, peanuts, pistachios, and chocolate	[47]
Pterostilbene	Stilbenes	blueberries, grape, and medicago sativa linn	[48]
Piceatannol	Stilbenes	grape, sugarcane, passion fruit, and blueberry	[49]

**Table 2:** List of dietary polyphenols exerting lipid-lowering effects via mTOR signaling.

Compound	Experimental Model	Functions and Mechanisms	Reference
Anhydroicaritin (5, 10 and 20 μM; 30 or 60 mg/kg)	HepG2 cells Western-type-diet mice	LKB1↑ → mTOR and P70S6K↓ → SREBPs↓ → lipid metabolism↑	[142]
Anthocyanins from Purple Corn (0.4 mg/mL)	3T3-L1 preadipocytes cells	mTOR, P70S6K and PKC↓ → insulin resistance↓	[143]
Betulinic Acid (1, 2, 3, or 4 μg/mL)	HepG2 cells	AKT↓ → mTOR↓ → S6K↓ → SREBPs↓ → de novo lipogenesis↓	[144]
Capsaicin (200 μM)	HepG2 cells	AMPK↑ → AKT↓ → mTOR↓ → SREBPs↓ → de novo lipogenesis↓	[145]
Curcumin (0.4 %/wt)	High-fat-diet mice	PI3K↓ → AKT↓ → mTOR↓ → NFкB↓ → colorectal cancer↓	[146]
EGCG (901.4 mg/d)	Obese female	mTORC2-; RICTOR-	[147]
Fisetin (50 μM; 0.2% or 0.5% (*w*/*w*))	3T3-L1 preadipocytes cells High-fat-diet mice	AKT↓ → TSC2↓ → S6K1 and mTOR↓ → C/EBPα and PPARγ↓ → adipogenesis↓	[148]
Fisetin (10 μM)	3T3-L1 preadipocytes cells	mTOR↓ → S6K↓ → C/EBPα↓ → GLUT4↓ → glucose uptake↓ → adipogenesis↓	[149]
Grape seed proanthocyanidins extracts (200 mg/kg)	High-fat-diet mice	mTOR↓ → adipogenesis↓ metabolism↑ → FOXO↓ → autophagy↓ → metabolic syndromes↓	[150]
Genistein (25 μM)	HepG2 cells	ERβ↑ → AKT and mTOR↓ → FASN and SREBPs↓ → lipogenesis↓; PPARα and CPT1↑ → fatty acid β-oxidation↑	[151]
Kaempferol (7.5, 15 and 30 μM)	3T3-L1 preadipocytes cells	AKT, mTOR and p70S6K↓ → C/EBPβ, KLF4 and KLF5↓, KLF2 and Pref-1↑ → PPARγ, C/EBPα and aP2↓ → lipid accumulation↓ → adipogenesis↓	[152]
Kaempferol (10 μM)	RIN-5F cells	PLN2↓ → lipid deposition↓; AMPK↑, mTOR↓ → LC3, p62 and Atg7↑ → lipophagy↑ → lipid stores↓	[153]
Lychee fruit extracts (20 or 200 mg/kg bw)	High-fat-diet mice	mTOR↓ → SREBPs↓ → lipogenesis↓	[154]
Oligonol (10, 25, and 50 μg/mL)	3T3-L1 cells	AMPK↓ → AKT↓ → mTOR↓ → p70S6K↓ → PPARγ and C/EBPα↓ → adipocyte differentiation↓ → adipogenesis↓	[155]
Oligonol (1, 5, and 10 μg/mL)	HepG2 cells	mTOR↓ → S6K↓ → insulin resistance↓	[156]
Pentamethylquercetin (20 mg/kg)	High-fat-diet mice	SIRT1↑ → mTOR↓ → 4EBP1↑ → autophagy↑; FAS, PPARγ, SREBPs↓ → adipogenesis↓	[157]
Penta-O-galloyl-α-D-Glucose (30 μmol/L)	3T3-L1 fibroblasts	mTOR↓ → PPARγ and C/EBPα↓; Pref-1↓, p21↑, cyclinD1↓ → G1 cell cycle arrest↑ → adipogenensis↓	[158]
Quercetin (6.25, 12.5 and 25 μM)	3T3-L1 preadipocytes cells	PI3K, AKT, mTOR and p70S6K↓ → PPARγ, C/EBPα and FABP4↓ → adipogenesis↓ → LPAATθ, DGAT1 and Lipin1↓ → lipogenesis↓	[159]
Resveratrol (0–100 μM)	RPE cells	pS6-→hyperinsulinemia↓	[160]
Resveratrol (200 mg/kg)	High-fat-diet mice	Lactococcus, Clostridium XI, Oscillibacter, and Hydrogenoanaerobacterium↓, Marinilabiliaceae and Turicibacter↑	[116]
Resveratrol (100 μM)	Primary preadipocyte	Akt↓ ⇄ Sirt1↑ → mTOR and S6K↓ → IL-6, MCP-1 and iNOS↓ → adipose inflammation↓;	[161]
Resveratrol (100 mg/kg)	High-fat-diet mice	AMPK↑ → mTOR↓ → p62↓, LC3↑ → autophagy↑	[162]
Resveratrol (25 μM)	L6 skeletal muscle cells	mTOR↓ → p70S6K↓ → IRS-1↑ → glucose uptake↓→insulin resistance↓	[163]
Sulforaphane (10 μM; 30 mg/kg)	Mouse fibroblast line 3T3-L1 pre-adipocytes High-fat-diet mice	AMPK↑ → mTOR↓ → ULK1↑ → LC3↑ → autophagy↑ → lipophagy↑	[164]

Notes: “↓” indicates down-regulation of expression or decrease of activity; “↑” indicates up-regulation of expression or increase of activity.

## Data Availability

Not applicable.

## Abbreviations

AMPK: AMP-dependent kinase; BAT: brown adipose tissue; C3G: cyanidin-3-O-β-glucoside; C/EBPα: CCAAT enhancer-binding protein alpha; CPT-1: carnitine palmitoyl transferase-I; EGCG: (−)-epigallocatechin gallate; HFD: high-fat diet; MCP-1: monocyte chemoattractant protein-1; MAPK: mitogen-activated protein kinase; mTOR: mechanistic target of rapamycin; mTORC1: mTOR complex 1; mTORC2: mTOR complex 2; PA: phosphatidic acid; PI3K: phosphoinositide 3-kinase; PPARα: peroxisome proliferator-activated receptor alpha; PPARγ: peroxisome proliferator activator receptor gamma; RHEB: ras homologue enriched in brain; SREBPs: sterol regulatory element binding proteins; S6K1: ribosomal protein S6 kinase 1; 4EBP1: inhibitory eIF4E-binding protein.
